# Evolving Threats: Adaptive Mechanisms of Monkeypox Virus (MPXV) in the 2022 Global Outbreak and Their Implications for Vaccine Strategies

**DOI:** 10.3390/v17091194

**Published:** 2025-08-30

**Authors:** Yuanwen Wang, Meimei Hai, Zijie Guo, Junbo Wang, Yong Li, Weifeng Gao

**Affiliations:** 1 College of Life Sciences, Ningxia University, Yinchuan 750021, China; 12023131021@stu.nxu.edu.cn (Y.W.);; 2 Key Laboratory of Conservation and Utilization of Special Biological Resources in Western China, Ministry of Education, Ningxia University, Yinchuan 750021, China

**Keywords:** zoonosis, monkeypox virus, immune escape, mechanism of infection, vaccine development

## Abstract

Monkeypox virus (MPXV) experienced an unprecedented global outbreak in 2022, characterized by a significant departure from historical patterns: a rapid spread of the epidemic to more than 110 non-traditional endemic countries, with more than 90,000 confirmed cases; a fundamental shift in the mode of transmission, with human-to-human transmission (especially among men who have sex with men (MSM)) becoming the dominant route (95.2%); and genetic sequencing revealing a key adaptive mutation in a novel evolutionary branch (Clade IIb) that triggered the outbreak. These features highlight the significant evolution of MPXV in terms of host adaptation, transmission efficiency, and immune escape ability. The aim of this paper is to provide insights into the viral adaptive evolutionary mechanisms driving this global outbreak, with a particular focus on the role of immune escape (e.g., novel mechanisms of M2 proteins targeting the T cell co-stimulatory pathway) in enhancing viral transmission and pathogenicity. At the same time, we systematically evaluate the cross-protective efficacy and limitations of existing vaccines (ACAM2000, JYNNEOS, and LC16), as well as recent advances in novel vaccine platforms, especially mRNA vaccines, in inducing superior immune responses. The study further reveals the constraints to outbreak control posed by grossly unequal global vaccine distribution (e.g., less than 10% coverage in high-burden regions such as Africa) and explores the urgency of optimizing stratified vaccination strategies and facilitating technology transfer to promote equitable access. The core of this paper is to elucidate the dynamic game between viral evolution and prevention and control strategies (especially vaccines). The key to addressing the long-term epidemiological challenges of MPXV in the future lies in continuously strengthening global surveillance of viral evolution (early warning of highly transmissible/pathogenic variants), accelerating the development of next-generation vaccines based on new mechanisms and platforms (e.g., multivalent mRNAs), and resolving the vaccine accessibility gap through global collaboration to build an integrated defense system of “Surveillance, Research and Development, and Equitable Vaccination,” through global collaboration to address the vaccine accessibility gap.

## 1. Introduction

Monkeypox virus (MPXV) is a double-stranded DNA virus belonging to the Orthopoxvirus family and is highly homologous to variola virus (VARV), with 96.3% amino acid sequence similarity [1]. Since May 2022, the monkeypox virus (MPXV) has entered a new phase of global transmission, with the outbreak affecting over 110 non-endemic countries and a cumulative total of over 90,000 confirmed cases reported [2]. During the 2024 PHEIC, cases in Africa accounted for over 80% of the total, with a significant increase in infection rates among children and a case fatality rate of 3–6%. Vaccination rates among key populations such as healthcare workers and sexually active individuals remained below 20% [3]. The Africa CDC estimates that at least 10 million doses of vaccine are needed to respond to the monkeypox outbreak dominated by the Congo Basin clade (Clade I). However, due to vaccine production capacity constraints and unequal distribution of international resources, vaccination rates in African countries are extremely low, resulting in significantly higher rates of severe illness and mortality compared to non-endemic regions [4]. It is worth noting that the proportion of human-to-human transmission has increased significantly during this epidemic. Epidemiological data shows that close contact transmission among men who have sex with men (MSM) accounts for as much as 95.2% of cases [5]. It shows that the virus might have evolved to spread more easily.

The smallpox vaccine provides 85% cross-protection against MPXV [6]. However, since the global cessation of smallpox vaccination in 1980, there has been a widespread lack of immunity to monkeypox virus. For example, in a research paper published on 19 January 2024, the research team led by Wang Jianwei and Guo Fei mentioned that the population born after 1980 showed almost no detectable antibodies, memory B cells, or memory T cell immune responses to the TianTan strain (VTT) of variola virus [7]. Research by the Zhang Zheng/Lu Hongzhou team also indicates that HIV-positive and HIV-negative individuals born after the cessation of smallpox vaccination lack vaccine-induced cross-protection against monkeypox virus [8]. This immunological gap in susceptible populations, combined with current insufficient vaccine coverage, poses a major public health risk [7].

MPXV was first discovered in 1958 in a group of crab-eating macaques in Copenhagen, Denmark. In 1970, the Democratic Republic of the Congo reported the first human case of monkeypox, involving an unvaccinated infant just nine months old [9]. Although the primary host of MPOX remains unknown, it is widely believed that primates (such as monkeys) and African rodents may transmit the virus to humans [10]. In 2021, the number of monkeypox cases reported in Nigeria increased by 300% compared to the average of the previous five years. This abnormal increase was the first indication that the monkeypox virus may have broken through its original geographical restrictions and had the potential for widespread transmission. The global epidemic in 2022 confirmed this—the monkeypox virus spread from Africa to other parts of the world and even established local transmission chains in some non-endemic areas [2]. This year, the global monkeypox epidemic is showing some new features, and gene sequencing has shown that the prevalent strains mainly belong to the IIb branch of monkeypox virus (MPXV) [11]. Viruses of this branch show strong adaptation during transmission, and there are multiple mutations in their genomes associated with host adaptation that may contribute to the spread of the virus in the population [12]. However, there is no clear evidence that these mutations directly lead to a significant increase in the efficiency of human-to-human transmission. In addition, the presence of multiple mutations in the OPG014 gene that lead to loss of function and that are present in all MPXV branches suggests that they may be related to long-term evolution and adaptation of the virus but are not markers of specifically enhanced transmission capacity.

Notably, Chinese researchers have made significant breakthroughs in the study of the M2 protein of the monkeypox virus. For example, the team led by Zhao Haiyan from the School of Life Sciences at Wuhan University and the team led by Deng Zengqin from the Wuhan Institute of Virology of the Chinese Academy of Sciences have revealed a new mechanism of immune evasion by the monkeypox virus M2 protein, which antagonizes the CD28/CTLA4-B7.1/2 signaling pathway by binding to the B7.1/2 co-stimulatory molecule, thereby inhibiting T cell activation [13]. It provides a key theoretical basis for vaccine target development and offers new insights into the defense against poxviruses.

The monkeypox virus (MPXV), which belongs to the Orthopoxvirus group, can easily enter host cells and multiply in the cytoplasm because of its special structure, large genome, and effective ways to avoid the immune system. Key gene mutations in the 2022 strain further enhanced its adaptability [12]. Elucidating the molecular mechanisms of immune escape in MPXV is the cornerstone for developing effective targeted antiviral therapies in the future. This paper also reviews the protective effects of existing vaccines, the development progress of new vaccines, and vaccination strategies. Through the analysis of multivalent vaccines and vaccine efficacy data, it provides scientific support for the construction of a precise prevention and control system.

## 2. Basic Biological Characteristics of Monkeypox Virus

### 2.1. Virus Structure and Classification

**Figure 1:**
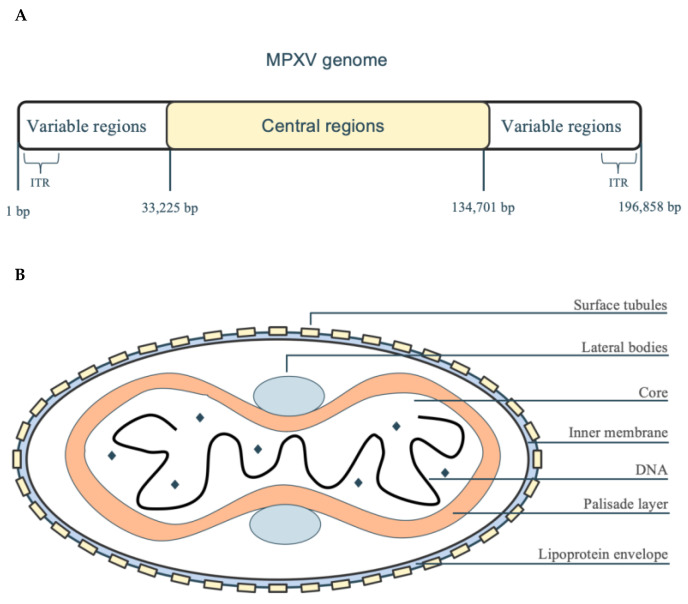
MPXV genome and structure. (**A**) The MPXV genome has a central part that is the same in all strains (134,701 bp) and is surrounded by two different parts (each 33,225 bp), with small, repeated sections (ITR, 1 bp) at both ends, making it about 196,858 bp long in total. The central region encodes essential genes for DNA replication and transcription, while the variable regions contain proteins associated with host adaptation and immune evasion. (**B**) The MPXV viral particle consists of a five-layered structure: a lipid protein envelope surrounding surface tubules and side bodies, a core containing the double-stranded DNA genome, and an outer palisade layer that maintains structural stability.

Monkeypox virus (MPXV) belongs to the Orthopoxvirus genus of the Poxviridae family, along with variola virus and cowpox virus [14]. MPXV viral particles are round but flattened, and they have a complicated structure that includes a fatty outer layer, surface tubes, two side bodies, and an inner double-curved core (Figure 1B) [15]. Glycoproteins such as A29, E8L, and M1R distributed on the outer membrane play an important role in the adsorption and membrane fusion of the host cell [16]; the core region contains viral DNA polymerase and transcription factors [17], driving viral genome replication and expression.

### 2.2. Genomic Characteristics

The MPXV genome is a linear double-stranded DNA molecule with a total length of approximately 196.8 kb (Figure 1A), making it one of the largest genomes within the Orthopoxvirus genus [18]. Its genome has inverted repeat sequences (ITR) at both ends, which form closed ring-shaped intermediates through palindromic structures during viral DNA replication, enhancing genome stability [19]. The central conservative region　encodes DNA polymerase (E9L) and DNA-dependent RNA polymerase subunit 18 (rpo18) [20], and the variable regions contain host range genes (such as CP77) and immune regulatory proteins (such as B6R and E8L) [21,22].

It is worth noting that the MPXV strain isolated during the global pandemic in 2022 belongs to evolutionary branch IIb [23]. In this evolutionary branch, the OPG027 gene underwent specific mutations. OPG027 is a gene that stops interferon, and it has three changes in its building blocks (I47V, F79L, and V126I) and one change that does not affect the protein (C276T). Among these, the F79L substitution is located at a critical hydrophobic site in OPG027. While I47V and V126I are located at two β-sheet regions of OPG027 [24], these mutations may affect MPXV’s transmissibility, pathogenicity, or immune evasion capabilities, serving as the molecular basis for enhanced human-to-human transmissibility. In-depth analysis indicates that the OPG027 protein (also known as C7L) is a key innate immune antagonist whose primary function is to inhibit the host type I interferon (IFN) response pathway [25]. The I47V and V126I mutations are located in the β-sheet region of the OPG027 protein, which is associated with protein stability, while the F79L mutation is located in the hydrophobic loop region of the OPG027 protein, which is highly conserved in poxviruses and directly involved in binding to the SAMD9 protein. Among these, the F79L mutation has the most significant impact on viral growth and host immune suppression, while the synergistic effects of all three mutations may enhance viral adaptability, replication efficiency, and pathogenicity. These mutations are associated with the high mortality rate of Clade I, reflecting the evolutionary pressure on MPXV in human populations [26]. This phenomenon highlights the importance of OPG027 as an anti-host immune target, providing new research perspectives for understanding MPXV’s evolutionary mechanisms, vaccine design (such as attenuated strategies), and antiviral interventions. Additionally, a systematic evolutionary analysis of the 2022 Clade IIb strain genome identified other mutations associated with viral adaptation, such as mutations in the outer membrane protein B21R (OPG210), which may enhance immune evasion and human-to-human transmission adaptability by altering the structure of surface glycoproteins, thereby driving the sustained outbreaks of this highly virulent branch [27]. The synergistic effects of these adaptive mutations collectively form the molecular basis for the rapid global spread of the IIb evolutionary branch.

### 2.3. The Immune Evasion Mechanism of the M2 Protein and Its Uniqueness in Viral Evasion Strategies

In addition to the mutations associated with innate immune evasion mentioned above, in terms of adaptive immune evasion, the multimeric structure of the M2 protein and its ability to bind to host B7 molecules with high affinity provide the virus with a powerful tool for directly inhibiting T cell activation. Regarding the immune escape mechanism, the team led by Zhao Haiyan from the School of Life Sciences at Wuhan University and the team led by Deng Zengqin from the Wuhan Institute of Virology of the Chinese Academy of Sciences resolved the structure of the complex between the monkeypox virus (MPXV) M2 protein and human B7.1 (hB7.1) and B7.2 (hB7.2) using cryo-electron microscopy [13]. The early secreted protein M2 encoded by monkeypox virus (MPXV) has been confirmed to act as a “molecular shield” against the host T cell response [28], with its escape strategy completed in three steps. First, high-order oligomerization: M2 rapidly assembles into hexamers or heptamers of a ring-shaped β-propeller structure (PDB ID: 8HAX/8HBX/8HCX) in the extracellular space. This conformation not only enhances stability but also amplifies the binding surface with target molecules; second, competitive occupancy: each M2 ring can simultaneously “capture” human B7.1 (CD80) and B7.2 (CD86) in a 6:6 or 7:7 molar ratio. The binding interface overlaps highly with the binding sites of T cell co-stimulatory receptors CD28/CTLA4 on B7 molecules, thereby physically blocking T cells from receiving the “second signal,” leading to the arrest of T cell activation. Cryo-EM further revealed that the interaction between M2 and B7.1 can also induce conformational changes in B7.1, enhancing its affinity for the inhibitory ligand PD-L1, forming a “double brake” that further suppresses the immune synapse; finally, functional validation: gene deletion experiments showed that the virulence of the M2-deficient strain was significantly reduced in primate models, suggesting that this mechanism is essential for sustained viral infection in vivo [13]. This finding provides a new perspective on understanding viral immune escape mechanisms and offers new insights for developing defensive strategies against monkeypox virus. Compared to common immune evasion strategies of other viruses, the mechanism of the MPXV M2 protein is more specific and direct:

The HIV-1 Nef protein downregulates MHC-I expression to avoid recognition by CD8^+^ T cells [29];

The CMV US2/US11 protein promotes the degradation of MHC-I [30];

The EBNA1 protein of EBV inhibits antigen presentation through Gly-Ala repeat sequences [31];

The E5/E6/E7 proteins of HPV interfere with interferon signaling and antigen processing [32].

Unlike these indirect or multi-step escape mechanisms, the M2 protein directly targets the second signal of T cell activation, thereby suppressing the adaptive immune response at an early stage. This mechanism is conserved in *Orthopoxvirus* but is rare in other viruses, providing a potential target for the development of specific antiviral drugs (such as inhibitors that block the M2-B7 interaction) [33].

## 3. The Infection Mechanism of Monkeypox Virus

Monkeypox virus (MPXV), a double-stranded DNA virus belonging to the Orthopoxvirus genus, has a complex and diverse infection mechanism. Recently, with the outbreak of the global monkeypox epidemic, researchers have gained a deeper understanding of MPXV’s infection mechanism. This chapter will explain how MPXV enters host cells, how it avoids the immune system, and how it interacts with the host, using the latest research to show how it has changed as it spread around the world.

### 3.1. Pathways of Monkeypox Virus Entry into Host Cells

Although the exact host and original source of MPXV remain unclear, the virus is known for its multiple transmission routes, including human-to-human, human-to-animal, and animal-to-animal transmission. Possible modes of transmission include respiratory secretions, saliva, skin lesions, sexual contact, and fecal contact [34]. The monkeypox virus undergoes initial replication at the site of entry into the skin or mucous membranes (such as broken skin, oropharynx, nasopharynx, etc.) [35]. Subsequently, the virus spreads to regional lymph nodes (such as lymphoid tissue in the neck and throat) and replicates extensively in the lymph nodes. It then spreads throughout the body via viremia, entering the bloodstream and spreading to other organs such as the spleen, liver, lungs, kidneys, intestines, and skin [36].

A key step in MPXV’s entering host cells is the binding of its surface spike protein to specific receptors on the host cell surface. For example, the spike protein can interact with molecules such as glycoproteins and heparan sulfate on host cells, thereby promoting viral endocytosis [37]. This process is crucial for the infection and transmission of the monkeypox virus. Research has found that the H3L protein made by the H3L gene of the monkeypox virus attaches to heparan sulfate on host cell surfaces, then enters the cell’s cytoplasm, and starts the replication process. Therefore, the H3L gene plays a crucial role in the process of viral particles invading host cells. H3L is an important target for vaccine development and the production of protective neutralizing antibodies [38]. In addition, the M2 protein of MPXV can specifically bind to hB7.1 and hB7.2, which are members of the B7 family of antigen-presenting cell surface proteins [13], and thus enter the host cell. The process by which viruses enter host cells not only depends on specific receptors but also involves multiple signaling mechanisms within the host cell, which collectively regulate viral replication and spread [39].

### 3.2. Molecular Mechanisms of Monkeypox Virus Replication, Pathogenesis, and Immune Evasion

Monkeypox virus has evolved multiple immune evasion strategies to escape host immune surveillance, ensuring its effective replication and transmission within the host. Recent studies have shown that MPXV encodes multiple immune evasion proteins that interfere with the host’s innate and adaptive immune responses.

**Figure 2:**
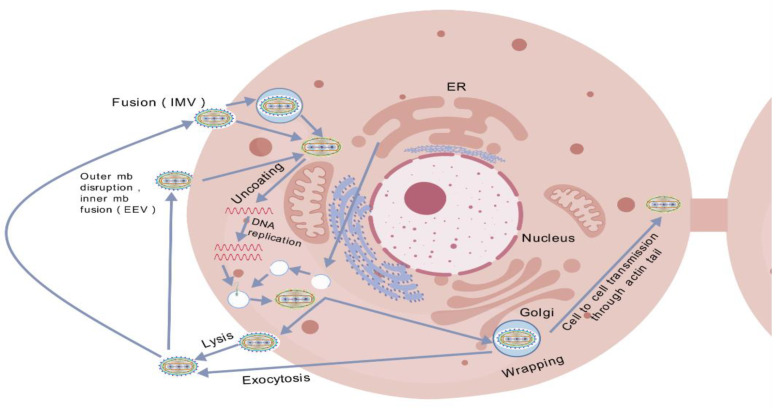
The life cycle of MPXV. Invade cells, replicate their genetic material, form new virions, and escape into the environment or other cells. (Note: EEV = enveloped virion; IMV = mature virion; ER = endoplasmic reticulum).

First, viral adhesion is mediated by the E8L protein, which binds to glycosaminoglycans (GAGs) on the surface of host cells, a critical step in viral entry into cells [40]. Therefore, disrupting this interaction can inhibit viral attachment. Monkeypox virus (MPXV) replicates its genome within the host cytoplasm using a DNA polymerase holoenzyme complex composed of F8 polymerase, processing factor A22, and uracil-DNA glycosylase E4, and forms “micronuclei” within the replication factory to protect viral DNA from detection by the host immune system [41,42]. The replication factories show how the virus takes advantage of the host cell’s processes, making it harder to create treatments that attack the virus without damaging the host cells. Figure 2 emphasizes the complexity of viral assembly, which involves different stages—crescent formation, immature virions, mature virions, and enveloped virions—that depend on various organelles [43]. This highlights the difficulty of coordinating the assembly process, which involves multiple cellular pathways—including endosomal trafficking, cytoskeletal rearrangement, and nuclear import—with the production of intracellular mature virions, thereby illustrating the complex nature of virion assembly rather than a singular biosynthetic step.

Monkeypox virus (MPXV) exhibits complex interactions with the host immune system to evade antiviral responses. It makes proteins like OPG027 and C7L that block T cell activation and control immune pathways like TNF, IL-17, and NF-κB, along with other immune pathways. These effects suppress the host’s antiviral activity, thereby achieving immune evasion. The virus also inhibits the expression of antiviral genes such as IFIT1 and IFIT2, which suppress viral mRNA translation, thereby counteracting the host’s defenses [44]. Proteins such as A49R are critical for replication and transcription, and specific mutations affect virulence and transmission [45]. The monkeypox virus adapts to human hosts through gene mutations such as S30L, D88N, and L108F. These mutations enhance viral replication, drug sensitivity, and host receptor binding capacity. The strain responsible for the 2022 outbreak exhibited mutations that increased the virus’s transmissibility and host interaction efficiency [12]. The impact on pathogenicity is particularly noteworthy. For example, functional studies have compared the virulence of the 2022 epidemic strain (Clade IIb) with that of the Central African branch (Clade I). Studies based on animal models indicate that while Clade IIb strains exhibit enhanced transmissibility, the severity of the disease they cause (e.g., extent of skin lesions, degree of systemic spread) may be lower than that of Clade I strains [46]. This virulence attenuation may be associated with mutations in certain genes, such as those related to host range (e.g., CP77). Adaptive evolution characterized by reduced virulence but enhanced transmissibility facilitates the establishment and maintenance of transmission chains in new host populations (global populations) [47], as it generates more asymptomatic or mildly symptomatic individuals capable of interacting with others, thereby significantly enhancing overall transmissibility. However, this trend is not absolute, and ongoing viral evolution must be closely monitored to prevent the emergence of variants with both high transmissibility and high pathogenicity. Although monkeypox virus replicates in the cytoplasm, it relies on host cellular machinery to synthesize viral proteins [48]. By reorganizing host cellular structures and encoding proteins such as anchor proteins and Bcl-2-like proteins (B5R and B17R), monkeypox virus ensures its efficient replication and immune evasion [36], and these complex molecular interactions have made the monkeypox virus a resilient pathogen and highlighted the need to develop targeted therapeutic strategies to disrupt its replication and immune evasion mechanisms.

M2 is a highly conserved immune escape protein in MPXV. In MPXV, M2 protein exists in a circular structure as a homomeric hexamer or heptamer. This multimeric form enhances its binding affinity (avidity) with hB7.1/2 [39]. The binding affinity of the M2 protein in its multimeric form to hB7.1/2 is significantly higher than that of the monomeric form, which is a key factor in its ability to effectively block the interaction between CD28 and hB7.1/2 [13]. M2 protein can specifically bind to hB7.1 and hB7.2, members of the B7 family, on the surface of antigen-presenting cells, while showing no significant interaction with other B7 family members (such as B7-H3, etc.). The place where M2 attaches to hB7.1/2 is very similar to where hB7.1/2 connects with the T cell surface receptors CD28/CTLA4, which means that the M2 protein can block the binding of CD28/CTLA4 to hB7.1/2. By attaching to hB7.1/2, M2 protein effectively prevents CD28 from interacting with hB7.1/2, which stops T cell activation. In vitro T cell activation experiments demonstrate that M2 protein can significantly inhibit hB7.1/2-mediated T cell activation, while the monomeric form of M2 protein, due to significantly reduced affinity for hB7.1/2, has almost lost its ability to inhibit T cell activation [13]. This further demonstrates the importance of M2 homopolymerization for its immune escape function.

In addition to the aforementioned mechanisms, several studies conducted following the global pandemic in 2022 have further identified several key proteins in monkeypox virus (MPXV) that are critical for enhancing its transmissibility and immune evasion capabilities. The A29L envelope protein on the surface of intracellular mature virus (IMV) is the primary target of monkeypox virus neutralizing antibodies. It plays a crucial role in viral recognition, fusion, and immune responses, and synergizes with the E8L protein to promote viral entry into host cells [48]. Chenguang Shen’s team obtained three monoclonal antibodies (9F8, 3A1, 2D1) by immunizing mice with MPXV A29L protein. Epitope identification and molecular docking analysis confirmed that they recognize different epitopes, and their synergistic antiviral activity against orthopoxviruses was validated through in vitro neutralization experiments and mouse models [49]. The M2 protein has emerged as a key immune evasion factor. Cryo-electron microscopy studies revealed that its hexamer/heptamer structure can bind with high affinity to human B7.1/B7.2 costimulatory molecules, effectively competitively inhibiting CD28/CTLA4 binding and thereby disrupting T cell activation—this newly discovered mechanism may explain the virus’s sustained transmission in human populations [50]. A research team led by Zhao Haiyan and Deng Zengqin from Wuhan University published a study in *Nature Communications* elucidating the immune evasion mechanism of the monkeypox virus M2 protein through inhibition of the T cell co-stimulation pathway, providing new insights for immunological intervention against poxviruses [13]. In terms of viral entry, the H3 protein mediates binding to heparan sulfate proteoglycans on the host cell surface, a critical first step in infection, facilitating viral adsorption and assembly, making it another promising vaccine candidate target [51]. The Nanjing University team used AI prediction (AlphaFold2), molecular dynamics simulation, and atomic force microscopy experiments to confirm that the 240–282 amino acids of the monkeypox virus H3 protein form a novel α-helix domain, which binds to host heparan sulfate through its positively charged amino acid clusters, initiating viral infection [52]. Additionally, the E8L protein interacts with glycosaminoglycans (GAGs) on the cell surface, and vaccine strategies targeting the disruption of this interaction are being explored [51]. A team from the University of Marseille in France used molecular docking and B-epitope prediction to identify the cyclic ganglioside-binding motif of the monkeypox virus E8L protein as a key target for viral adsorption and proposed a novel vaccine strategy based on this finding [39]. The synergistic actions of these proteins—A29L/E8L and H3 are responsible for attachment/entry, M2 for immune suppression—provide multifaceted strategies for MPXV to effectively establish infection in new human hosts. Summarizing these findings is crucial for developing targeted antiviral drugs and next-generation vaccines that can effectively block these specific virus–host interactions.

### 3.3. The Impact of Monkeypox Virus Immune Scape Mechanisms on the Infection Process

The monkeypox virus has numerous genes involved in immune evasion functions such as viral recognition, apoptosis, and immune regulation. In addition to having immune evasion proteins similar to those found in other poxviruses that control different signaling pathways, the monkeypox virus has its own unique ways to avoid the immune system that are different from those of the vaccinia virus. When the monkeypox virus infects various human cells, such as macrophages, fibroblasts, and HeLa cells in vitro, it selectively inhibits the expression of host genes that play important roles in activating innate immune response signaling pathways, such as interferon-induced genes, and can replicate normally even in the presence of type I interferon (IFN-α). Subsequent studies revealed that the type I interferon-binding protein (IFNα/βBP) encoded by monkeypox virus binds to the host cell membrane to inhibit the type I interferon signaling pathway [53]. In addition, although monkeypox virus infection induces NK cell expansion in rhesus monkeys, it significantly inhibits NK cell killing function [54]. Unlike variola virus and vaccinia virus, monkeypox virus can also escape immune recognition and response by CD4+T cells and CD8+T cells inhibiting T cell activation [55]. This may be used to promote the spread of the virus within the infected host. It was subsequently discovered that the monkeypox virus-encoded 197 and D14L proteins play an important role in inhibiting T cell function [56]. These studies indicate that the monkeypox virus has evolved multiple mechanisms to evade host innate and adaptive immune responses.

The immune evasion mechanisms of monkeypox virus significantly influence the course of infection and clinical manifestations. First, by inhibiting T cell and type I interferon signaling pathways, monkeypox virus can avoid rapid immune clearance by the host, thereby prolonging the survival time of the virus in the body [57]. Secondly, the monkeypox virus also regulates the expression of cell cycle and cycle-related genes to provide an optimal environment for viral replication [58]. Finally, this immune escape strategy not only increases the virus’s ability to spread but may also lead to severe clinical symptoms such as lymphadenopathy, rash, and multi-organ damage [36].

In summary, monkeypox virus (MPXV) achieves effective transmission and infection within the host through immune evasion strategies. However, two key scientific questions remain to be clarified: first, the molecular regulatory pathways underlying the phenotypic transition from genome replication to particle assembly during the viral lifecycle have not yet been fully elucidated; second, the regulatory mechanisms by which host cell-specific factors influence viral replication fidelity and immune evasion efficacy require systematic analysis. Overcoming these scientific bottlenecks will provide a theoretical foundation for developing precise, targeted therapeutic strategies. Therefore, learning more about these molecular processes will help explain how MPXV causes disease and will lead to new antiviral treatments that focus on important points in the interactions between the virus and the host.

## 4. Research Progress on Monkeypox Virus Vaccines

The global outbreak of monkeypox virus (MPXV) has made vaccine development a key strategy for controlling the epidemic. As the monkeypox epidemic spread in 2022, research institutions around the world accelerated their efforts to develop vaccines against MPXV. This chapter will systematically review the protective effects of existing vaccines, the development progress of new vaccines and vaccination strategies, and discuss the future direction and challenges of vaccine development in light of the latest research findings.

### 4.1. Protective Effects of Existing Vaccines

Currently, vaccines targeting monkeypox virus primarily rely on the cross-protective effects of smallpox vaccines. Smallpox vaccines (such as ACAM2000) have approximately 85% cross-protection against monkeypox virus [59]. For example, ACAM2000, a second-generation smallpox vaccine with a long history and strong immunogenicity, has been approved by the US Food and Drug Administration (FDA) for the prevention of monkeypox virus infection [60]. However, since the World Health Organization declared the eradication of smallpox in 1980, smallpox vaccinations have been discontinued worldwide, resulting in a widespread lack of cross-immunity to monkeypox among people under the age of 40 [61].

With the same ingredients as JYNNEOS, the safer MVA-BN vaccine has been approved by the EU and Canada for use in adults, and in 2024 the WHO approved it for use in adolescents aged 12–17 [4]. During the 2022 monkeypox outbreak, Bavarian Nordic, a Danish company, developed the JYNNEOS vaccine, a recombinant non-replicating vaccine based on modified Ankara vaccinia virus (MVA-BN) targeted against smallpox and monkeypox viruses. It has a high safety profile and is suitable for high-risk groups such as immunocompromised individuals and pregnant women. Its efficacy has been validated in animal studies and during the 2022 outbreak, with a post-exposure preventive efficacy of 88.8% against monkeypox [62]. Two doses of the JYNNEOS vaccine are effective in preventing a large number of monkeypox cases. For example, a study of 43 jurisdictions in the United States found that people who received only one or two doses of the JYNNEOS vaccine had a significantly lower risk of contracting monkeypox compared to those who were not vaccinated [63].

Among the two attenuated vaccines for monkeypox available on the market, ACAM2000 and JYNNEOS, as listed in Table 1, the latter was the primary vaccine used during the monkeypox outbreak in the United States, providing rapid protection for high-risk populations. However, both vaccines have certain limitations. For example, attenuated virus vaccines inevitably pose certain safety concerns in immunocompromised individuals, and the supply of the JYNNEOS vaccine is also very limited [64]. Therefore, the market urgently needs vaccines that are quick to develop, highly safe, and provide complete protective immunity.

**Table 1:** Statistical table of monkeypox virus vaccines.

Vaccine Name	Research and Development Institutions/Companies	Vaccine Type	The Research and Development Phase	Protective Effect	Approval Status	Note
MVA-BN	Danish non-replicating vaccine	Attenuated live vaccine	Approved for use	Highly effective protection	Approved	Used to prevent monkeypox, widely used worldwide [65].
ACAM2000	American smallpox vaccine	Attenuated live vaccine	Approved for use	Highly effective protection	Approved	Based on the smallpox vaccine, it has a protective effect against monkeypox [4].
MVA strain monkeypox attenuated live vaccine	Sinopharm Group	Attenuated live vaccine	Phase I clinical trial	To be verified	Approved for clinical trials in 2024	The first batch of participants for Phase I will be enrolled by January 2025, marking China’s first monkeypox vaccine to enter clinical trials [66].
LC-16	Japan’s minimum replication vaccine	Attenuated live vaccine	Approved for use	High	Approved	Based on the smallpox vaccine, it has a protective effect against monkeypox [67].
VTT	Sinopharm Group	Viral vector vaccine	Unspecified	High	Widely used in China from 1950 to 1969	Long-term and large-scale use in China [68,69].
VGPox1-3	Sinopharm Group	mRNA vaccine	Preclinical stage	Animal experiments show excellent immune protection effects	Not approved	World’s first monkeypox mRNA vaccine containing a combination of M1R and A35R antigens [70].
BNT166	BioNTech	mRNA vaccine	BNT166 Clinical Assessment Conducted (NCT05988203)	Preclinical experiments protect mice and monkeys	Not approved	Polyvalent design, tetravalent vaccine (A35, B6, M1, H3); trivalent vaccine (without H3) [71].
VGPox	Multivalent mRNA vaccine developed by the Seventh Affiliated Hospital of Sun Yat-sen University	mRNA vaccine	Preclinical stage	To be verified	Not approved	mRNA vaccines against monkeypox and other poxviruses [70].
JYNNEOS	JYNNEOS vaccine in the United States	Attenuated live vaccine	Approved for use	Highly effective protection	Approve	For the prevention of monkeypox, based on the smallpox vaccine [72].

### 4.2. Development of New Vaccines

With the rapid development of mRNA vaccine technology, several companies have begun to develop monkeypox vaccines based on the mRNA platform. Moderna’s mRNA-1769 vaccine has shown excellent protective efficacy in animal models. This is an mRNA-LNP vaccine targeting the MPXV surface protein [4]. It can significantly reduce viral load and the severity of lesions, and no severe or extremely severe infection cases were observed in non-human primate animal experiments. Additionally, the antibodies produced in animals vaccinated with the mRNA-1769 vaccine demonstrated superior binding capacity to viral proteins compared to the current mainstream vaccine JYNNEOS (i.e., the modified Ankara cowpox virus vaccine), indicating superior immune efficacy. In lethal primate models it outperformed MVA-BN, with stronger antibody responses and faster symptom resolution [4]. This vaccine uses mRNA lipid nanoparticle (LNP) technology to encode four key proteins (A29, A35, B6, and M1) on the surface of the monkeypox virus. These proteins are crucial for viral replication and are highly conserved. Experiments have shown that after vaccination, animals not only produced neutralizing antibodies but also induced a broad immune response, including functional antibodies and cross-immunity protection [73]. Moderna’s mRNA-1769 vaccine will enter Phase I/II clinical trials in 2024 [4]. It is expected to become a more efficient new type of vaccine. BioNTech has also launched the BNT166 project, which aims to develop a multivalent mRNA vaccine to address mutations in the monkeypox virus. The BNT166 vaccine has shown good protective effects in preclinical studies, completely protecting mice and rhesus monkeys from monkeypox virus infection [69].

In the development of new vaccines, the Mix series of multivalent vaccines were designed by screening monkeypox virus (MPXV) surface proteins (including MVs and EVs), including Mix-4 (4 antigens), Mix-8 (8 antigens), and Mix-12 (12 antigens). Research indicates that the Mix-12 vaccine demonstrates the best performance in terms of neutralizing antibody titers and protective efficacy. Its multivalent design significantly enhances antibody diversity, enabling it to address viral mutations [62]. The vaccine also significantly reduced the risk of viral escape. The MPX-EPs vaccine targeting cellular immunity activates CD8+T cells by enriching CTL epitopes (containing ubiquitin modification) and independently induces cellular immune protection [62]. The study found that the vaccine significantly reduced viral load in the lungs of mice in a passive immunization model, demonstrating strong cellular immune protection [62]. Further synergistic immune strategies combine the Mix-12 and MPX-EPs vaccines, achieving synergistic effects between humoral immunity (neutralizing antibodies) and cellular immunity (CTL) through co-administration. This co-administration approach provides complete protection against high-dose MPXV infection, fully demonstrating the potential of mRNA vaccines in infectious disease control [62].

Currently, monkeypox vaccine development focuses primarily on viral vector vaccines (such as MVA) and mRNA vaccines. Although DNA vaccines have been studied, they have not yet become the mainstream approach to monkeypox prevention and control [62].

### 4.3. Comparative Analysis of Vaccine Platforms: Advantages, Disadvantages, and Applicable Scenarios

The global outbreak of MPXV has accelerated the development of various vaccine platforms, each with unique characteristics. A comparative analysis of these platforms is crucial for formulating reasonable vaccination strategies tailored to specific epidemiological contexts and resource environments. In the following sections, we will systematically evaluate the advantages, limitations, and primary application scenarios of the main vaccine technologies, as shown in Table 2.

In summary, the selection of a vaccine platform requires a balance between immunogenicity, safety, deployment speed, and logistical feasibility. Currently, viral vector vaccines such as MVA-BN, with their proven efficacy and acceptable safety profile, serve as a cornerstone of global response strategies [74]. mRNA vaccines, with their unparalleled development speed and ability to adapt to viral evolution, represent a transformative platform and an ideal choice for addressing future variants and outbreaks [75]. Live attenuated vaccines, despite their strong immunogenicity, are restricted to specific applications due to safety concerns [76]. Looking ahead, a heterogeneous vaccine strategy leveraging the strengths of each platform, tailored to target populations and epidemic dynamics, is crucial for achieving sustainable control of MPXV.

**Table 2:** Comparison of mainstream vaccine platforms.

Evaluation Dimensions	Viral Vector Vaccines (e.g., MVA-BN, JYNNEOS)	mRNA Vaccines (e.g., mRNA-1769, BNT166)	Attenuated Live Vaccines (e.g., ACAM2000, LC16)	Protein Subunit Vaccine (Under Development)
Immunogenicity	Induces strong humoral and cellular immunity, supported by long-term real-world data (~85% efficacy) [77].	Highly immunogenic, demonstrating superior neutralizing antibody and T cell responses compared to MVA-BN in preclinical studies [78].	Highest immunogenicity, capable of inducing very strong and persistent immunity [77].	Immunogenicity is highly dependent on adjuvants, is usually weak, and requires multiple immunizations [79].
Safety	High safety (non-replicating), suitable for immunocompromised individuals [80].	High safety, no viral vectors, no integration risk [81].	Low safety, with risks of spreading cowpox, myocarditis, encephalitis, etc. Not suitable for immunocompromised individuals [77].	Highest safety, no genetic material, no risk of infection [82,83].
	The platform is mature, but production depends on cell culture, which takes a long time.	Extremely fast research, development, and production, making it easy to respond to virus mutations and pandemics [84].	The platform is mature, but production is complex and the cycle is long [74].	Long R&D cycle, production requires protein expression and purification, slow speed.
Responding to variability	Based on conserved antigens, providing cross-protection [75].	Can respond to mutations and rapidly design and produce multivalent vaccines targeting new variants [76].	Provides broad-spectrum protection, but may become ineffective once antigenic drift occurs [85].	Can be designed for specific antigens, but must be redesigned to address mutations, and is slower than mRNA [86].
Applicable scenarios	Large-scale vaccination, emergency reserves, and preferred choice for immunocompromised populations [74].	Responding to new outbreaks and variants, rapid deployment, an ideal choice as a booster shot [75].	Emergency vaccination for children, to be used when medical resources are sufficient [87].	Suitable for people with extreme safety requirements (such as those who are highly allergic to vaccine ingredients) [88].

### 4.4. Vaccination Strategy

The formulation of vaccination strategies requires consideration of vaccine efficacy, safety, and the needs of the target population. First, there are limitations to existing strategies, as there is an insufficient supply of approved vaccines (such as MVA-BN and LC16m8) [3]. This makes it extremely difficult to cope with the surge in cases in Africa. Secondly, vaccine distribution is driven by commercial interests, with high-income countries hoarding vaccines and low-income countries facing shortages, leading to barriers to equitable access and high costs. Africa’s reliance on imported vaccines exacerbates this imbalance, as it produces only 1% of the world’s vaccines, severely limiting its ability to respond to the pandemic [89].

To address the issues of uneven vaccine distribution and low vaccination rates, it is imperative to establish a multi-dimensional and feasible implementation framework that seamlessly integrates global collaboration with localized strategies.

First, global coordination and financing mechanisms should be strengthened. It is recommended that, under the coordination of the World Health Organization (WHO), the Global Alliance for Vaccines and Immunization (Gavi), and the Coalition for Epidemic Preparedness Innovations (CEPI), a MPXV vaccine access pool be established for monkeypox, utilizing a transparent allocation framework that prioritizes distribution based on disease burden (such as the number of cases per capita) rather than purchasing power [90]. This mechanism requires binding procurement commitments from high-income countries to ensure the sharing of funds and doses. For example, multiple countries have called for enhanced vaccine sharing, with China and others donating and sharing vaccines through bilateral agreements and the COVAX mechanism during the pandemic [91]. Second, substantial technology transfer and localized production in Africa must be promoted [92]. The mRNA vaccine technology transfer center established by the World Health Organization in South Africa aims to support vaccine production and technical training in African countries, but its actual effectiveness and progress remain controversial [93]. BioNTech’s collaboration with Rwanda and Senegal demonstrates Africa’s vaccine production potential, but this has not yet been realized on a large scale [94]. Bavarian Nordic, the manufacturer of the JYNNEOS vaccine, has not yet explicitly authorized African countries to use its production technology, but it possesses certain technical capabilities in vaccine production [95].

At the regulatory and logistics levels, efforts should be made to promote mutual recognition among African Union member states for WHO prequalified vaccines, accelerate import procedures, and invest specifically in cold chain infrastructure [96]. The experience of Gavi’s Cold Chain Equipment Optimization Platform (CCEOP) in deploying solar refrigerators in the Democratic Republic of the Congo and Nigeria could be leveraged and explicitly expanded to include the storage and transportation of MPXV vaccines [97].

In terms of vaccination implementation, a tiered strategy should be adopted: single-dose ACAM2000 for emergency post-exposure prophylaxis in emergency or resource-limited areas, then two-dose JYNNEOS as the standard preventive regimen under routine conditions, with post-exposure vaccination clearly required to be completed within one week of exposure [4]. Additionally, public education should be strengthened to reduce stigma and vaccine hesitancy, and high-risk groups (such as healthcare workers, immunocompromised individuals, and children) should be prioritized for vaccination to maximize vaccine protection [98,99].

Through these specific and mutually supportive measures, the challenges of monkeypox vaccine distribution and administration can be fundamentally addressed, transforming equitable access from a concept into a feasible reality.

### 4.5. Analysis of the Protective Efficacy and Global Coverage of Existing Vaccines

The issue of insufficient global monkeypox vaccine coverage has become a key bottleneck in epidemic prevention and control. People who were vaccinated against smallpox before 1980 have cross-protection against monkeypox (approximately 85% protection rate), but with the decline of immunity, the protective effect has significantly decreased in people under the age of 40. JYNNEOS (MVA-BN) vaccine is a replication-deficient modified vaccinia Ankara virus vaccine. It was approved by the FDA in 2019 for the prevention of smallpox and monkeypox and received prequalification from the WHO in 2024, enabling it to prevent both smallpox and monkeypox. Clinical trials have shown that the vaccine is approximately 85% effective in preventing monkeypox. It is suitable for high-risk individuals aged 18 and above (such as laboratory workers, healthcare workers, and close contacts). The vaccine is administered in two doses, with a 28-day interval between doses. The combined efficacy of the two doses can reach 90% [100]. A 2022 study by the US CDC showed that post-exposure vaccination with JYNNEOS can reduce the risk of infection by 70–80% (must be administered within four days of exposure) [10]. The smallpox vaccine is currently out of use, and JYNNEOS is being used on an emergency basis during the monkeypox outbreak [63]. This vaccine works by inducing neutralizing antibodies against the poxvirus genus (such as smallpox and monkeypox), with the main target being the viral surface protein VP37. Although there are antigenic differences between monkeypox and smallpox, cross-immunity remains effective, particularly in post-exposure prophylaxis [10]; this vaccine is suitable for people with immune deficiencies. ACAM2000 is a replicating smallpox vaccine that replaced Dryvax in 2007. Its efficacy against monkeypox is comparable to that of JYNNEOS [10], but the side effects are more pronounced (such as myocarditis, encephalitis, and skin infection risks) [62]. ACAM2000 is not suitable for use by people with immune suppression, pregnant women, or people with allergies. The APSV vaccine, a replicating vaccine, is available only under Emergency Use Authorization (EUA) or Investigational New Drug (IND) authorization [10]. There is also a live attenuated vaccine developed in Japan that has cross-protective potential against monkeypox. Animal models show that its protective effect is comparable to that of MVA-BN, but its global coverage is low and it is mainly used in Japan [100].

From 2022 to 2024, 110 countries worldwide reported monkeypox outbreaks, with MSM populations accounting for over 90% of infections and vaccine coverage in low-income countries below 10% [62]. High-coverage regions include the Americas and Europe, where countries such as the United States and Brazil have reduced transmission rates by 75% through large-scale vaccination campaigns. In the United Kingdom and Germany, vaccination rates for priority groups (MSM, healthcare workers) have exceeded 80%. Low-coverage regions include Africa and Asia, where only 12% of high-risk populations in the Democratic Republic of the Congo were vaccinated in 2024, constrained by inadequate cold chain infrastructure and supply shortages. In India and Southeast Asian countries, vaccination coverage remains below 5%. Some countries rely on donated vaccines [100]. Key factors contributing to this uneven coverage include the limitations of existing vaccines such as the limited production capacity of the JYNNEOS vaccine, with a global annual production capacity of approximately 30 million doses, which is insufficient to meet the demand during large-scale outbreaks. Another factor is the restricted use of ACAM2000 due to its potential for serious side effects, with the WHO recommending its use only in regions with adequate medical resources [62]. Supply inequality, such as the fact that 90% of vaccines worldwide were concentrated in high-income countries in 2023, is contributing factor, with the COVAX mechanism prioritizing the distribution of COVID-19 vaccines to low-income countries while the distribution of monkeypox vaccines lagged behind [10]. Awareness and stigma, such as 30–50% of MSM refusing vaccination due to privacy concerns, and cold chain requirements, such as MVA-BN needing to be stored at −20 °C, limit distribution in resource-poor areas [101]. Price and production capacity are also contributing factors: the cost of a single dose of JYNNEOS is approximately US$100, which is a heavy burden for low- and middle-income countries to bear when vaccinating on a large scale [10].

### 4.6. Breakthrough Progress in China’s Independently Developed Vaccines

The attenuated live vaccine developed by the Shanghai Institute of Biological Products and other institutions is in the IND stage, but no data on its protective efficacy has been released yet [3]. Three mRNA vaccines developed by Sinopharm, VGPox1-3, have entered the clinical trial stage. Their protective efficacy is yet to be verified, and they have not been approved for market release [77]. The multivalent mRNA vaccine developed by the Seventh Affiliated Hospital of Sun Yat-sen University is about to enter the clinical trial stage. In challenge experiments, it has also shown excellent results, protecting mice 100% from monkeypox virus infection. It has not yet been approved for market release [102].

### 4.7. Challenges and Future Directions

Current vaccines rely on cross-protection from traditional smallpox vaccines. Existing vaccines (such as ACAM2000 and MVA-BN) were originally designed for smallpox and offer cross-protection against monkeypox (e.g., MVA-BN showed 78% efficacy in England), but their protective efficacy may be insufficient [103,104]. Furthermore, coverage is insufficient, with non-epidemic countries (such as Europe and the United States) receiving priority access to vaccines while epidemic areas in Africa (such as the Democratic Republic of the Congo) have low vaccination rates, and resource-limited regions face challenges with cold chain transportation [104]. Secondly, there is the issue of safety. First-generation vaccines (such as ACAM2000) contain live viruses and may cause serious side effects (such as myocarditis), making them unsuitable for immunocompromised individuals [77]. Although third-generation vaccines (such as MVA-BN) are relatively safe, there is still insufficient data on their use in pregnant women and newborns [105]. Existing vaccines are designed to target historical strains, and their efficacy against currently circulating MPXV lineages (such as Clade IIb) requires further validation [106]. Most candidate vaccines are still in the animal testing phase and lack human data. Fluctuations in the monkeypox epidemic have made it difficult to recruit participants for clinical trials, affecting the assessment of efficacy [107]. Finally, there is the threat of new variants. The MPXV clade (formerly Clade IIb) that emerged in 2022 has increased transmissibility. However, existing vaccines are primarily based on Clade II, so their protective efficacy against Clade I is limited [101].

In the future, target optimization will be conducted for the development of a monkeypox-specific vaccine, focusing on MPXV-specific antigens (such as L1R, B5R, A27L and A33R) and viral particles (IMV/EEV), with the aim of enhancing protection through multivalent design [76]. Then, a new DNA vaccine platform will be established. For example, the 4pox vaccine has shown effects comparable to MVA-BN in animal models, but clinical validation is required [77]. A new mRNA vaccine platform will be created: multivalent mRNA vaccines can induce T cell responses and effectively combat poxviruses in animal experiments [108]. A novel protein subunit vaccine platform will also be established, with adjuvanted composite antigen vaccines in the preclinical stage [109]. Responding to viral evolution requires testing for genetic mutations in MPXV to ensure the conservatism of vaccine targets. For instance, we should develop broad-spectrum vaccines for different clades (Clade I/II) [106]. To address global accessibility, vaccination procedures need to be simplified (e.g., single-dose vaccination) and costs reduced to promote widespread use in low- and middle-income countries [103]. WHO prequalification expands access to MVA-BN in low-income countries [110]. Finally, heterologous booster vaccination (such as initial vaccination with a DNA vaccine + booster vaccination with an mRNA vaccine) will be explored to enhance the immune response [111]. A new universal vaccine (such as mRNA technology) targeting multiple branches (Clade I/II) should also be developed [112].

There are currently three vaccines approved worldwide for the prevention of monkeypox: the Danish non-replicating vaccine (MVA-BN), the Japanese minimally replicating vaccine (LC16), and the US-based variola-derived replicating vaccine (ACAM2000). These vaccines are all based on attenuated live virus smallpox vaccines. A retrospective study by the WHO showed that smallpox vaccination is 85% effective in preventing monkeypox [113]. Domestic vaccines have not yet been approved for market release, but the development of attenuated live vaccines and mRNA vaccines is progressing rapidly, with several entering the clinical trial phase. mRNA vaccines have become a hot topic in research and development due to their ability to rapidly respond to mutations. A Chinese team has developed the world’s first monkeypox mRNA vaccine [3]. Currently available vaccines (ACAM2000/JYNNEOS/LC16) are all derived from smallpox vaccines and have safety or supply limitations. Multivalent vaccine designs (such as VGPox1-3) can address viral mutations and represent a future trend [70].

## 5. Conclusions and Outlook

Monkeypox virus (MPXV), as a zoonotic pathogen, presents major problems for public health systems due to its global spread and the complexity of its infection mechanisms. The global outbreak of MPXV has highlighted the complexity of the spread of emerging infectious diseases in a globalized context. This study integrates genomic epidemiology and vaccine efficacy data to draw the following conclusions: (1) The evolutionary branch differentiation of MPXV (Clade I/II) and key gene mutations (such as OPG027 and M2) significantly enhance its transmissibility and immune evasion capabilities, with Clade IIb becoming the dominant strain in non-endemic regions; (2) Vaccination is a core control measure, but existing vaccines (JYNNEOS, ACAM2000) lack sufficient protective data against Clade I, and global distribution disparities exacerbate control challenges in high-burden regions like Africa; (3) China’s vaccine development progress is rapid, with multivalent mRNA vaccines potentially overcoming technical limitations of traditional vaccines, but clinical trials and market approval processes need to be accelerated.

Future research directions should focus on the following: (1) Virus evolution monitoring and functional validation: Establish a global real-time MPXV genome tracking network to provide early warnings for potentially highly transmissible/highly pathogenic variants. Specific research should include the following: establishing standardized, high-throughput monitoring protocols to identify emerging mutations in key immune escape proteins such as OPG027 and M2 in real time; utilizing reverse genetics systems to conduct gain-of-function studies on critical mutations identified through monitoring (e.g., F79L in OPG027) to clarify their specific roles in viral adaptation, virulence, and immune evasion; and developing AI-based predictive models integrating genomic sequences and clinical data to assess transmission risks and vaccine escape potential of new variants. (2) Precision optimization of vaccine strategies: Develop broad-spectrum vaccines targeting Clades I/II and explore single-dose vaccination regimens. Corresponding research objectives should include the following: design and evaluate multivalent mRNA vaccines covering clade-specific antigens (e.g., simultaneously expressing A29L, H3, B6R, and M2 proteins) to elicit cross-neutralizing antibodies and cellular immune responses against viruses from different lineages; evaluate the protective efficacy of single-dose vaccination regimens in non-human primate models, clarifying the intensity and persistence of induced immune memory responses to inform herd immunity strategies in resource-constrained regions; and conduct clinical trials to investigate heterologous booster strategies (e.g., MVA primary vaccination followed by mRNA booster), assessing their potential to overcome waning immunity from smallpox vaccination and induce stronger, broader immune responses. (3) Multidimensional prevention strategy efficacy evaluation: Combining behavioral interventions, screening, and diagnostic technologies to interrupt transmission chains. Future research should conduct prospective cohort studies to quantify the actual effectiveness of behavioral interventions (e.g., digital health education targeting MSM populations) in reducing infection rates; validate the sensitivity and specificity of novel rapid detection technologies (e.g., CRISPR or antigen rapid test kits) in border control and community settings to establish scalable early diagnosis protocols; and develop dynamic transmission models using hospital data to assess the cost-effectiveness of integrated “detection-alert-isolation” strategies in curbing hospital-based transmission and community outbreaks. (4) Global collaboration mechanisms to narrow vaccination coverage gaps require moving beyond pledges to actionable, funded initiatives: Launch a MPXV vaccine access pool to ensure equitable distribution based on public health needs; scale up successful technology transfer models—such as BioNTech’s collaboration with Rwanda and the WHO’s mRNA hub in South Africa—to incorporate MPXV vaccines and foster regional self-reliance; strengthen continental regulatory coordination through the African Medicines Agency (AMA); and invest in tailored logistics solutions (e.g., Gavi’s CCEOP) to overcome distribution challenges. These steps, proven effective in other health crises, offer a viable pathway to vaccine equity. Therefore, addressing the challenge of a prolonged MPXV epidemic requires building a closed-loop prevention and control system integrating surveillance, R&D, vaccination, and treatment.
